# Classification of genetic variants in genes associated with Lynch syndrome using a clinical history weighting algorithm

**DOI:** 10.1186/s12863-016-0407-0

**Published:** 2016-07-01

**Authors:** Brian Morris, Elisha Hughes, Eric Rosenthal, Alexander Gutin, Karla R. Bowles

**Affiliations:** Myriad Genetics, Inc, 320 Wakara Way, Salt Lake City, UT 84108 USA; Myriad Genetic Laboratories, Inc, 320 Wakara Way, Salt Lake City, UT 84108 USA

**Keywords:** Lynch syndrome, *MLH1*, *MSH2*, *MSH6*, Variant classification

## Abstract

**Background:**

Lynch syndrome is a hereditary cancer syndrome associated with high risks of colorectal and endometrial cancer that is caused by pathogenic variants in the mismatch repair genes (*MLH1*, *MSH2*, *MSH6*, *PMS2*, *EPCAM*). Accurate classification of variants identified in these genes as pathogenic or benign enables informed medical management decisions. Previously, we developed a clinical History Weighting Algorithm (HWA) for the classification of variants of uncertain significance (VUSs) in *BRCA1* and *BRCA2*. The *BRCA1/2* HWA is based on the premise that pathogenic variants in these genes will be identified more often in individuals with strong personal and/or family histories of breast and/or ovarian cancer, while the identification of benign variants should be independent of cancer history. Here we report the development of a similar HWA to allow for classification of VUSs in genes associated with Lynch syndrome using data collected through both syndrome-specific and pan-cancer panel testing.

**Methods:**

Upon completion of algorithm development, the HWA was tested using simulated variants constructed from 79,214 probands, as well as 379 true variants. Positive (PPV) and negative predictive values (NPV) were calculated on a per gene basis.

**Results:**

25,500 pathogenic and 50,500 benign simulated variants were analyzed using the HWA and the PPVs and NPVs for each gene were greater than 0.997 and 0.999, respectively. The HWA was also evaluated using 100 trials for each of the 379 true variants. PPVs of >0.998 and NPVs of >0.999 were obtained for all genes.

**Conclusions:**

We have developed and implemented a HWA to aid in the classification of VUSs in genes associated with Lynch syndrome. The work presented here demonstrates that this HWA is able to classify *MLH1*, *MSH2*, and *MSH6* VUSs as either benign or pathogenic with high accuracy.

## Background

Lynch syndrome (LS) is an autosomal dominant hereditary cancer syndrome associated with high risks of colorectal cancer (CRC) and endometrial cancer (EC). While US population CRC and EC risks to age 70 are estimated to be 5 % and 2.6 %, respectively, individuals with LS have much higher risks of up to 82 % and 71 % [1, 2]. LS is also associated with increased risks for gastric, urothelial, ovarian, small bowel, pancreatic, brain, and sebaceous gland cancers [1]. LS is caused by pathogenic variants in the DNA mismatch repair (MMR) genes, *MLH1, MSH2, MSH6,* and *PMS2,* as well as deletions in *EPCAM* which affect the transcription of *MSH2* [1, 3]. It is important to identify individuals who carry pathogenic MMR gene variants so that they can be targeted for aggressive medical management interventions aimed at the prevention and/or early detection of LS-associated malignancies [2].

As is the case for many of the genes responsible for hereditary cancer syndromes, analysis of the LS-associated MMR genes frequently results in the identification of variants that can reliably be predicted to result in loss of function (LOF) of the encoded protein due to premature truncation or other significant structural disruptions. These LOF variants are presumed to be pathogenic, and causative of LS [4]. However, it is also common to identify genetic variants of uncertain clinical significance (VUSs), for which pathogenicity cannot be assumed. Examples include missense variants altering a single amino acid, changes for which the impact on mRNA splicing cannot be reliably predicted, changes in known/suspected promoter regions or the 5’-untranslated region, and in-frame insertions or deletions of small numbers of amino acids. In the majority of cases, it is not possible to establish the clinical significance of VUSs without collecting and analyzing large amounts of clinical data linked to testing outcomes in patients and families. This process can take years, or even decades, particularly in the case of rare variants.

Although the identification of a VUS in a LS gene is not diagnostic of LS and should not be used as a basis for medical management, a VUS result creates uncertainty and anxiety that cannot be completely resolved until a definitive classification is established. Individuals carrying VUSs which are actually pathogenic may not receive appropriate medical care until the classification is established, exposing them to risks that might otherwise have been mitigated. Therefore, it is vitally important to expand the tools available for accurate and timely variant classification.

The need for improved variant classification tools has become more urgent as hereditary cancer genetic testing is increasingly performed with large panels of genes, or even entire exomes, rather than smaller subsets of genes associated with individual conditions like LS. The use of panels including genes for multiple hereditary cancer syndromes has already demonstrated that pathogenic mutations in LS-associated genes are frequently identified in individuals who might not have been ascertained for LS testing based on their personal and family histories of CRC and EC [5, 6]. While this validates the benefits of a broader pan-cancer panel approach to testing, it is inevitable that analysis of more genes leads to the identification of more VUSs.

We have previously described the development and validation of a clinical History Weighting Algorithm (HWA) designed to aid in the reclassification of variants identified in the *BRCA1* and *BRCA2* genes, which are associated with Hereditary Breast and Ovarian Cancer syndrome (HBOC) [7]. The *BRCA1/BRCA2* HWA is based on the premise that pathogenic variants in these genes will be identified more often in individuals with strong personal and/or family histories of breast and/or ovarian cancer, while the identification of benign variants should be independent of cancer history. We report here on the development of a HWA to allow for classification of VUSs in LS-associated genes using data collected through both syndrome-specific and pan-cancer panel testing.

## Methods

The development and implementation of the HWA was performed as previously described for the *BRCA1* and *BRCA2* genes with relevant exceptions noted below [7].

### Patient ascertainment

All patients were over the age of 18 and underwent clinical genetic testing from a Clinical Laboratory Improvement Amendments (CLIA) and College of American Pathology (CAP) approved laboratory. Informed consent for testing and results interpretation was obtained. Clinical information was collected on the test request form. No additional information was obtained directly from patients or providers for this study and all clinical testing samples used for algorithm development and testing were anonymized. As such, this study was not subject to any additional ethics board review.

After informed consent, healthcare providers collected patient blood, buccal or saliva samples for comprehensive sequencing analysis of the *MLH1, MSH2* and *MSH6* genes. Analysis may have either been limited to these three genes, or these genes may have been incorporated into a larger 25 gene panel [5]. Large rearrangement analysis and/or analysis of *PMS2* may or may not have been performed. The following clinical information was collected: proband age, ethnicity, and cancer history, including cancer type(s) and age(s) of diagnosis (if applicable). Affected relative cancer histories, including cancer type(s) and age(s) of diagnosis were also documented. Patient clinical conditions included in analysis were colorectal, endometrial and/or ovarian cancers. Patients affected with other, rare Lynch syndrome-associated cancers, including hepatic, gastric, hepatobiliary, pancreatic, small bowel, and brain, were excluded from analysis due to limited available data. Patients affected with cancers unrelated to Lynch syndrome were included; however, any unrelated cancers/conditions were not considered in HWA development. Probands were excluded from analysis if personal and/or family history were not provided or if the proband was known to carry a pathogenic mutation or VUS in a Lynch-associated gene, in addition to the variant being analyzed.

### History weighting score calculation and analysis

For each patient in the dataset, the probability of carrying a pathogenic mutation, conditional on personal and family history, was estimated from observed proportions in a clinical population and stored in gene-specific conditional probability tables, as previously described [7]. For each proband, a likelihood ratio was calculated as the ratio of the posterior odds to the prior odds of the proband not carrying a pathogenic mutation. Assuming independence, these likelihood ratios (LR) were cumulatively multiplied for probands carrying the same variant to obtain a history weighting score (HWS) for the variant (HWS = (LR_1_)(LR_2_)…(LR_n_)). For variants with a large number of proband observations, only the most recent 100 probands were analyzed.

The variant-specific HWS was then used in a two-hypothesis test: Two empirical cumulative distribution functions (ECDF) were constructed from the logs of the HWS of probands carrying either known pathogenic mutations in the gene of interest (positive control ECDF) or from probands in whom Lynch syndrome pathogenic mutations and/or VUS in any disease-associated gene were not identified (negative control ECDF) [7]. The variant-specific HWS was compared against each ECDF.

In the previous implementation, a minimum number of qualifying proband observations of a variant was required for utilization of the HWA. Minimum numbers were specific to the gene of interest and the type of call being attempted (“pathogenic” or “benign”). ECDFs were generated from 100,000 positive or negative composite control variants, which were constructed as previously described [7]. Briefly, for each proband carrying a specific variant, a minimum of 100 unique positive control individuals, known to carry a pathogenic variant in the gene of interest and matched by ethnicity and time of testing, were selected. Controls were initially limited to individuals tested within ±180 days of the proband’s test date. If there were insufficient ethnically “matched” controls identified within the ±180 window, ethnically “unmatched” controls from within the same window were utilized, as time of testing ascertainment biases were more significant than ethnicity biases (data not shown). Ethnically “unmatched” controls were also used for individuals not specifying ancestry. If control numbers were still insufficient, the window was expanded by another ±180 days, iteratively. Benign (negative) control probands were similarly selected from individuals carrying either benign or no variants. Each composite control variant, represented in the ECDFs by its corresponding history weighing score, was composed of the same number of control probands as the variant of interest. The HWA made a benign call if the variant-specific HWS was greater than the 99.95th percentile of the positive control ECDF, and greater than the 5th percentile of the negative control ECDF. The HWA made a pathogenic call if the variant-specific HWS was less than the 0.05th percentile of the negative control ECDF, and less than the 95th percentile of the positive control ECDF.

The current implementation used gene-specific thresholds defined in terms of standard deviations of the control ECDFs and reduced the number of ECDF composite control variants from 100,000 to 10,000. The HWA made a benign call if the variant-specific HWS was greater than the 99.5th percentile plus some number of standard deviations of the positive control ECDF, and greater than the 1st percentile of the negative control ECDF. The HWA made a pathogenic call if the variant-specific HWS was less than the 0.5th percentile minus some number of standard deviations of the negative control ECDF, and less than 99th percentile of the positive control ECDF (Table 1).

**Table 1:** Classification thresholds utilized by the history weighting analysis tool

Classification call	Log history weighting score compared to pathogenic composite controls	Log history weighting score compared to benign composite controls
Pathogenic	<99th Percentile	<0.5 Percentile - 2.2/2.3/2.2 SD
Weak Pathogenic^a^	≥99th Percentile and ≤ 99.5 Percentile + 0.7/0.9/1.0 SD	<0.5 Percentile - 2.2/2.3/2.2 SD
Not Callable	>99.5 Percentile + 0.7/0.9/1.0 SD	<0.5 Percentile - 2.2/2.3/2.2 SD
Weak Benign^a^	>99.5 Percentile + 0.7/0.9/1.0 SD	≥0.5 Percentile - 2.2/2.3/2.2 SD
		≤1st Percentile
Benign	>99.5 Percentile + 0.7/0.9/1.0 SD	>1st Percentile
Indeterminate	All Other Variants	

Gene-specific standard deviations (SD) are indicated for *MLH1, MSH2* and *MSH6,* respectively

^a^Pathogenic and benign ECDF control curve thresholds must not overlap

Gene and call type-specific standard deviation numbers were established by four two-fold cross validations (8 folds total) utilizing the full dataset of 79,214 probands and analysis of simulated variants, which were constructed as previously described [7]. Briefly, assuming a pathogenic simulated variant composed of *n* individuals, *n* gene-specific variants were chosen uniformly at random from true pathogenic variants. Then, one individual was randomly chosen from each selected variant. These *n* individuals were combined to simulate a single pathogenic variant. In order to simulate unknown relatedness between probands, each proband after the first selected proband was randomly duplicated with a 0.05 probability. Similar methodology was used to construct benign simulated variants.

For each cross validation, the proband dataset was divided randomly in half. Half A was used to construct conditional probability tables, and simulated variants, as described by Pruss et al*.* [7], were constructed from Half B (and *vice-versa*). A minimum number of five qualifying proband observations was required regardless of gene or attempted call type. Pathogenic mutation prevalence estimates of 24.2 %, 16.8 % and 12.9 % (data not shown) were used for *MLH1, MSH2,* and *MSH6*, respectively. Positive (PPV) and negative (NPV) predictive values were adjusted for prevalence as previously described for each gene and call type with only pathogenic and benign calls being counted towards PPVs and NPVs. Indeterminate calls were excluded from additional analysis. It was assumed that pathogenic mutations in the same gene confer identical risks.

### Testing with true variants

One hundred trials were performed for each variant. For each variant-specific trial, five probands carrying the variant of interest were randomly selected and an algorithm call attempted. If an algorithm call was successful, the variant was scored for that trial as either pathogenic or benign. If there was insufficient data for a call, the proband number was increased by 20 % and another call attempted. This process was repeated until a call was successful or all available probands were utilized. Previously defined standard deviation thresholds were utilized for analysis. PPVs and NPVs were calculated.

## Results

### Determination and verification of classification thresholds

The HWA was developed and tested using a dataset of 79,214 probands who had undergone clinical genetic testing for mutations specifically in Lynch syndrome-associated genes or who had opted for testing utilizing a 25-gene pan-cancer panel [5]. The accuracy of the HWA was dependent on the establishment of classification thresholds, which were utilized by the calculation to call a variant pathogenic or benign. In this study, two types of thresholds were utilized, one based on pre-established ECDF percentiles and the other based on standard deviations from these ECDF percentiles (Table 1). Standard deviation thresholds were pre-determined using four two-fold cross validations (8 folds in total) performed on simulated variants. Standard deviation classification thresholds resulting in average PPVs and NPVs of >0.9975 across all 8 cross-validation folds and with no predictive values less than 0.996 were selected for algorithm implementation. These threshold values were similar for each gene, ranging from 0.7 to 1.0 when downgrading variants to 2.2 to 2.3 when upgrading variants. However, standard deviation numbers required to upgrade a variant to pathogenic were more than double those required to downgrade a variant to benign. This was due to the high *a priori* probability that a particular variant was most likely benign, which increased the likelihood of a false positive when a pathogenic call was made.

Once classification thresholds were established, additional testing with two-fold cross validation of the conditional probability tables was performed utilizing simulated variants. PPVs and NPVs, adjusted for prevalence, were greater than 0.996 and 0.997 for each gene, respectively (Table 2).

**Table 2:** Results of two-fold cross validations using simulated variants

		Fold A		Fold B	
*MLH1*		Variant type		Variant type	
		Pathogenic	Benign	Pathogenic	Benign
HWA Call	Pathogenic	24880	60	24836	35
	Benign	176	49748	166	49765
	No Call	444	692	498	700
Total Trials		25500	50500	25500	50500
PPV		0.9962		0.9978	
NPV		0.9978		0.9979	
*MSH2*		Variant Type		Variant Type	
		Pathogenic	Benign	Pathogenic	Benign
HWA Call	Pathogenic	24775	12	24262	14
	Benign	111	50329	164	50243
	No Call	614	159	1074	243
Total Trials		25500	50500	25500	50500
PPV		0.9988		0.9986	
NPV		0.9991		0.9987	
*MSH6*		Variant Type		Variant Type	
		Pathogenic	Benign	Pathogenic	Benign
HWA Call	Pathogenic	23787	9	24014	25
	Benign	180	50262	88	49754
	No Call	1533	229	1398	721
Total Trials		25500	50500	25500	50500
PPV		0.9987		0.9965	
NPV		0.9990		0.9995	

### Proband numbers required for classification calling

**Fig. 1:**
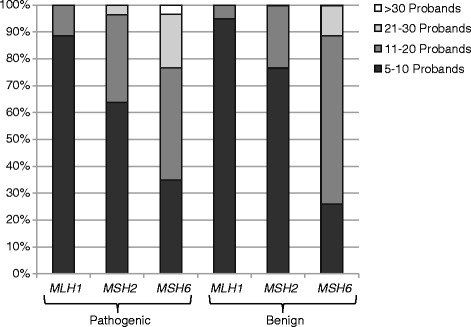
Relationship between the proband number required to make an HWA classification call, gene and call type (pathogenic or benign). Fewer probands were required for the classification of *MLH1* and *MSH2* variants in comparison to *MSH6* variants. Generally, fewer probands were also required for classification of benign variants in comparison to pathogenic variants for the same gene

Conditional probability tables were constructed using the entire dataset of 79,214 probands, and 25,500 pathogenic and 50,500 benign simulated variants were constructed for each gene. Simulated variants were analyzed using the HWA and the number of probands required to make a call were documented for each gene and variant type (Fig. 1). Fewer probands were required to make a pathogenic or benign variant call for the *MLH1* and *MSH2* genes in comparison to the *MSH6* gene due to the relatively higher penetrance of these two genes, allowing for easier separation of control ECDFs. For a specific gene, fewer probands were required for a benign call versus a pathogenic call as the *a priori* probability that a variant was benign was significantly higher than that of a variant being pathogenic. PPVs and NPVs for each gene were greater than 0.997 and 0.999 for each gene, respectively (Table 3).

**Table 3:** Results of final testing using simulated variants

*MLH1*		Variant type	
		Pathogenic	Benign
HWA Call	Pathogenic	25162	17
	Benign	61	50150
	No Call	277	333
Total Trials		25500	50500
PPV		0.9989	
NPV		0.9992	
*MSH2*		Variant Type	
		Pathogenic	Benign
HWA Call	Pathogenic	24904	29
	Benign	69	50306
	No Call	527	165
Total Trials		25500	50500
PPV		0.9971	
NPV		0.9995	
*MSH6*		Variant Type	
		Pathogenic	Benign
HWA Call	Pathogenic	25038	6
	Benign	23	50322
	No Call	439	172
Total Trials		25500	50500
PPV		0.9992	
NPV		0.9999	

Conditional probabilities tables were constructed based on the entire dataset of 79,214 probands

### True variant trials

**Fig. 2:**
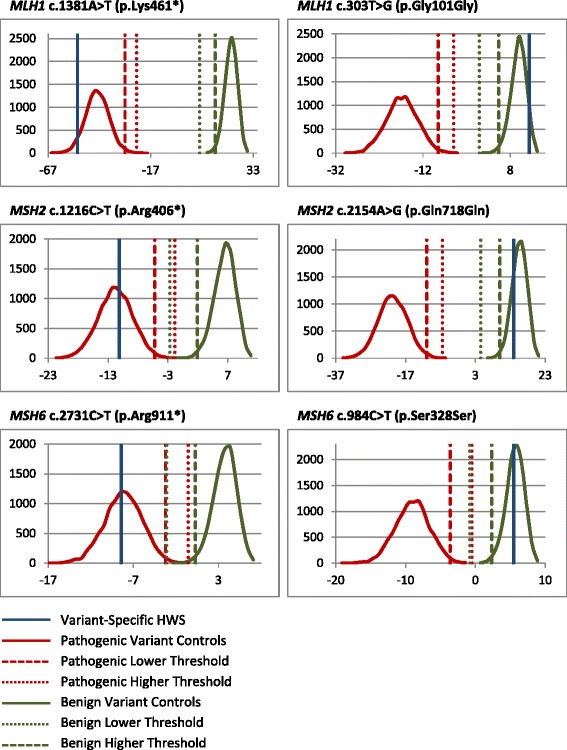
HWA graphs illustrating classification calls for select representative variants. The x-axis of each graph indicates the log of the history weighting score and the y-axis indicates the number of control variants

379 anonymized true variants which had been previously identified through clinical testing and classified using other methodologies were used to assess the accuracy of the HWA. Due to limited availability of variants with sufficient proband numbers for HWA analysis, probands were selected from the same dataset used for HWA development. 100 trials were performed for each variant. PPVs of >0.998 and NPVs of >0.999 were obtained for all genes (Table 4). Results of representative variants for each gene and variant call type are provided (Fig. 2).

**Table 4:** Testing results for true variants (100 trials per variant)

*MLH1*		Variant type	
		Pathogenic	Benign
HWA Call	Pathogenic	4890	2
	Benign	7	4328
	No Call	1403	1370
Total Trials		6300	5700
PPV		0.9986	
NPV		0.9995	
*MSH2*		Variant Type	
		Pathogenic	Benign
HWA Call	Pathogenic	2877	1
	Benign	1	5388
	No Call	822	2011
Total Trials		3700	7400
PPV		0.9991	
NPV		0.9999	
*MSH6*		Variant Type	
		Pathogenic	Benign
HWA Call	Pathogenic	1906	1
	Benign	0	6955
	No Call	1694	4244
Total Trials		3600	11200
PPV		0.9989	
NPV		100.0000

## Discussion

Timely and accurate reclassification of VUSs to more definitive clinical categories is critical for patient care, but gathering and interpreting the necessary clinical and biological evidence can be a time-consuming and resource-intensive process. As illustrated by the work described here for the LS genes, the HWA provides a robust, accurate and quantitative tool applicable to a relatively high proportion of variants, complementing other traditional classification tools, each of which has significant limitations. It is particularly important to remain aware of these limitations now, as the shift to testing strategies based on large gene panels, as well as whole exomes/genomes, creates pressure to quickly resolve the classification of the large number of variants identified. Failing to correctly identify pathogenic variants deprives patients of the opportunity to utilize appropriate options for lowering the risk of disease. Conversely, incorrectly classifying benign changes as pathogenic can result in the misapplication of interventions which may carry significant risks, morbidities and unnecessary costs.

Many limitations of traditional variant classification techniques are addressed by the HWA method. Unlike segregation analysis, the HWA does not rely on testing multiple relatives from large pedigrees, and since it pools data from multiple families, it is less likely to result in the misclassification of a VUS due to its linkage to a different, undetected pathogenic change in a subset of carriers. Additionally, HWA is a more direct measure of a variant’s clinical significance than other classification techniques, including assays to measure mRNA splicing analysis or protein function, such as mismatch repair in the case of the LS genes. These analyses assume that the measured functional effect directly translates to a clinical outcome, but this is not always the case, and it is often unclear how to interpret situations where only partial loss of function is observed. The HWA more directly measures a variant’s clinical impact by determining the variant’s relationship to the relevant personal/ family histories of carriers.

Algorithm development is often limited by access to larger datasets of variants with definitive classifications. For example, attempting to develop a classification algorithm using a dataset composed of previously classified missense variants requires the assumption that all of the prior classifications are indeed correct. Since the HWA is agnostic to variant type (i.e. missense, nonsense, splicing, silent, etc.), we were able to develop the model using probands carrying variants with relatively unambiguous classifications. Pathogenic proband controls were largely composed of individuals carrying truncating variants (i.e. nonsense and frameshift) or canonical splice junction variants, which are generally assumed to be pathogenic. Probands with pathogenic variants of other types were included in this control set only if additional highly significant data supported a pathogenic classification. Likewise, negative control probands either carried no known variants in any Lynch syndrome gene or carried variants which are assumed to be benign. Most of these benign variants were not predicted to result in an amino acid change or were present in the general population at a frequency too high to be causative of Lynch syndrome. The ability to use a dataset composed of variants with “clear cut” classifications is ideal for algorithm development. Utilizing this dataset, we were able to extend a HWA validated for classification of variants in the *BRCA1* and *BRCA2* genes to the LS genes *MLH1*, *MSH2,* and *MSH6*.

An additional advantage of HWA is that it can be used for variants with relatively modest numbers of proband observations (Fig. 1). HWA classification thresholds used in the initial implementation for *BRAC1* and *BRCA2* were relatively rigid in that they utilized hard ECDF control percentiles as cut-offs, and a minimum number of probands was required before a classification call was attempted [7]. While this approach resulted in high accuracy, it sometimes unnecessarily penalized variants with lower numbers of proband observations. Previously, we had repeatedly observed variants that did not have the minimum number of probands required to be eligible for analysis (data not shown). However, HWA scores for these variants were so extreme, that inclusion of the additional required probands statistically could not have resulted in a different classification call made by the HWA. The modification of the ECDF thresholds used in this current study permits classifications with significantly lower proband numbers, 5 being set as the minimum required for analysis. For *MLH1* and *MSH2*, >96 % of pathogenic or benign variant calls could be made with ≤ 20 proband observations of the specific variant, and >63 % of calls could be made with ≤ 10 proband observations. The relatively small number of probands required positions the HWA as a powerful tool for the classification of both common and relatively rare variants, in the context of high volume clinical testing. For lower testing volume settings or extremely rare variants, other techniques may still be necessary for the reclassification of VUS.

It is extremely difficult to identify low penetrance variants with current variant classification methods, and the HWA shares this limitation. Our previously described experience with known hypomorphic variants in *BRCA1* and *BRCA2* has shown that, even after observations in large numbers of probands, these lower penetrance variants will not produce a HWS falling within the defined threshold for either the negative or positive control ECDF.^7^ Variants behaving in this fashion are clearly suspect for lower penetrance, but we cannot rule out the possibility that there are some LS gene variants called as benign with the HWA which are associated with low penetrance for one or more Lynch cancers. It is arguable as to whether this type of variant should be regarded as pathogenic for LS, since current medical management guidelines for LS would probably not apply.

An additional consideration regarding the HWA is the advantage conferred by utilizing data from only a single high volume laboratory. Documentation of clinical history can be influenced by multiple factors, including the design of the test requisition form, criteria used to ascertain patients for testing, payor coverage criteria, provider specialty, the structure of the healthcare system, patient characteristics, etc. These factors will inevitably vary between laboratories. Basing the HWA on data from a single laboratory increases the consistency of the data used to generate a variant’s HWS matched to the negative and positive control ECDFs, and it facilitates the identification of related probands. Efforts to apply the HWA to pooled data from multiple sources would most likely either not be possible or would require significantly higher thresholds and proband numbers to guard against incorrect classifications.

## Conclusions

We have developed and implemented a history weighting algorithm to aid in the reclassification of VUS in Lynch syndrome genes to more definitive clinical categories, promoting improved patient care and better clinical outcomes. The high accuracy of the HWA makes this classification technique the gold standard for reclassification of *MLH1, MSH,* and *MSH6* VUS in the clinical diagnostic setting. Additional modifications of the HWA may allow this tool to be extended to other autosomal dominant high and moderate risk genes responsible for increased cancer risk.

## Abbreviations

CRC, colorectal cancer; EC, endometrial cancer; ECDF, empirical cumulative distribution function; HBOC, hereditary breast and ovarian cancer; HWA, history weighting algorithm; HWS, history weighting score; LOF, loss of function; LR, likelihood ratios; LS, Lynch syndrome; MMR, mismatch repair; NPV, negative predictive value; PPV, positive predictive value; VUS, variant of uncertain significance
